# Lime and lithiasis: a case of choledocholithiasis complicated by limy bile

**DOI:** 10.1093/jscr/rjae245

**Published:** 2024-04-26

**Authors:** Travis Fahrenhorst-Jones, Jane E Theodore, Ratna Aseervatham

**Affiliations:** Division of Surgery, Princess Alexandra Hospital, Woolloongabba, QLD 4102, Australia; Department of Surgery, University Hospital Geelong, Barwon Health, Geelong, VIC 3220, Australia; Department of Surgery, Sunshine Coast University Hospital, Birtinya, QLD 4575, Australia

**Keywords:** limy, bile, jaundice

## Abstract

Limy bile syndrome (LBS) is a condition in which the biliary tract is filled with radiodense calcium carbonate rich sludge. This rare condition can complicate the management of commonly encountered biliary conditions such as choledocholithiasis. We present a case of a male in his fifties who presented to hospital with a 12-day history of abdominal pain, nausea and jaundice. Imaging and laboratory findings demonstrated a dependent radio-dense substance within the biliary system as well as an obstructing calculus at the duodenal ampulla. Management with endoscopic retrograde cholangiopancreatography alone was insufficient and further surgical management was required. With no clear published guidelines on LBS and associated cholelithiasis, management is variable. We present this case as an addition to the literature on the management of choledocholithiasis complicated by LBS.

## Introduction

Most frequently diagnosed incidentally in the context of symptomatic cholelithiasis, limy bile syndrome (LBS) is a condition rarely encountered by surgeons, gastroenterologists and radiologists alike. There currently exists no single guideline for the management of LBS and awareness of the condition among clinicians remains limited based on published case reports. Endoscopic and surgical techniques are the mainstay of treatment for LBS. We present this case of choledocholithiasis complicated by limy bile as an addition to the limited literature concerning this rare condition.

## Case report

A man aged in his 50s was referred to the emergency department with painful obstructive jaundice. The patient reported 12 hours of worsening epigastric pain on a background of 12 days of associated nausea, vomiting and anorexia. Two days before, he noticed a yellow discolouration of the skin, without changes to the colour of his urine or stool. The patient experienced no fevers, sweats or rigors, nor exposure to questionable foods or recent travel.

This presentation was on a background of a similar episode eight days prior. Resolution of pain, normal liver function tests (LFTs) and equivocal ultrasound imaging meant he was subsequently discharged.

Past medical history included a right sided kidney stone 27 years prior and regular colonoscopies for surveillance due to a family history of colon carcinoma. The patient consumed two standard drinks of alcohol per week.

On examination the patient was jaundiced. Observations were within normal limits. His abdomen was soft with mild right upper quadrant tenderness, no palpable mass and negative Murphy’s sign. Blood results were as per Table 1.

**Table 1:** Blood results at presentation

**Parameter**	**Value**	**Reference Range**
Total Bilirubin	104 mmol/l	<20 mmol/l
Conjugated Bilirubin	65 mmol/l	<4 mmol/l
Alkaline phosphatase	235 mmol/l	30-110 mmol/l
Gamma-glutamyl transferase	464 mmol/l	<55 mmol/l
Alanine transaminase	560 mmol/l	<45 mmol/l
Aspartate transaminase	312 mmol/l	<35 mmol/l
Lipase	48 u/l	20–300 u/l
Parathyroid Hormone	2.7 pmol/l	1-7 pmol/l
Corrected Calcium	2.3 mmol/l	2.1-2.6 mmol/l

**Figure 1:**
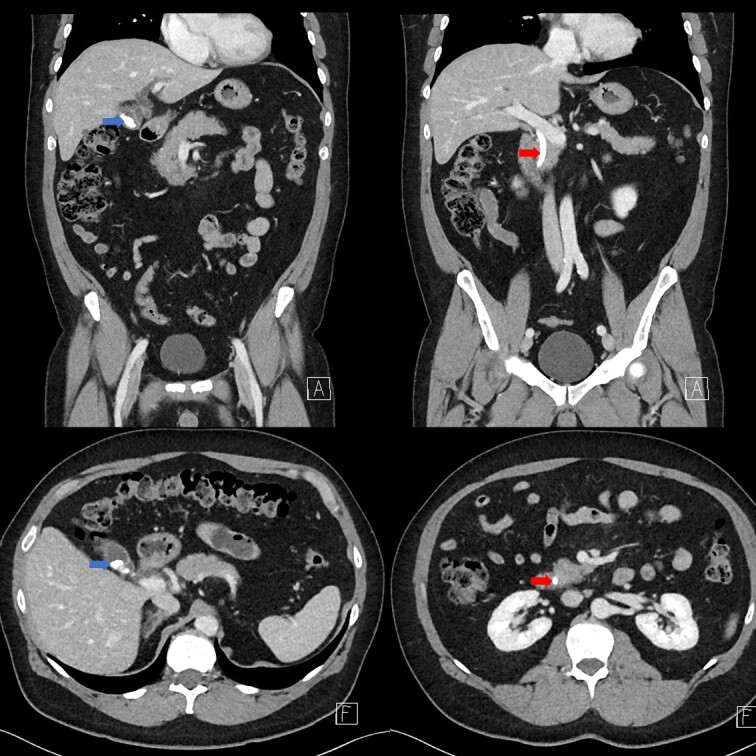
Contrast-enhanced CT demonstrating limy bile within the gallbladder neck (left image) and CBD (right image).

**Figure 2:**
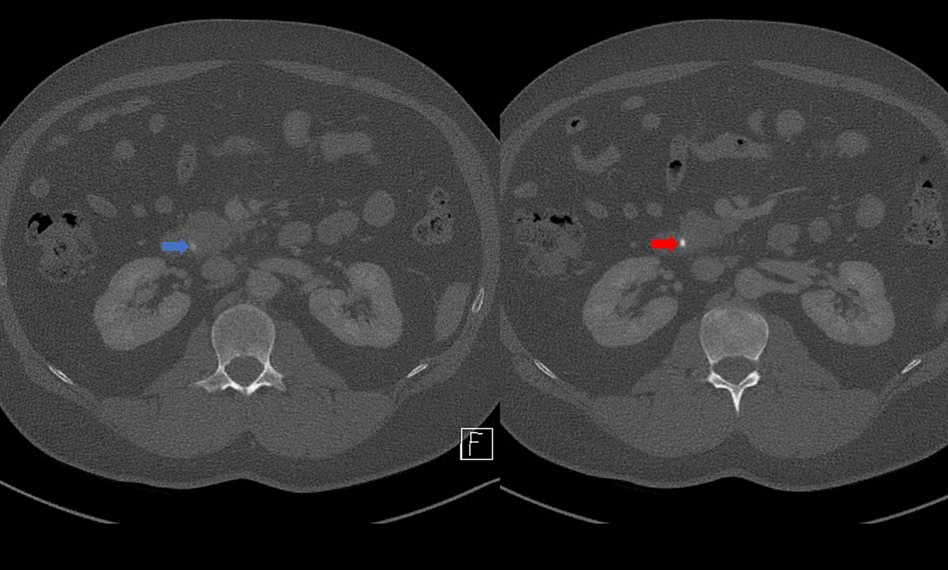
CT demonstrating an obstructing calculus (right image) present within the CBD distinct from the limy bile (left image) (WW 2249, WL 450).

Portal venous contrast enhanced computed tomography (CT) of the abdomen revealed a layered dependent hyperattenuating material within the biliary tree (Fig. 1). The common bile duct (CBD) was dilated measuring 7.5 mm in diameter and filled along its course with radiodense material. Mild distention of the cystic duct and intrahepatic biliary tree were also noted with a 4 mm calculus in the CBD at the level of the duodenal ampulla (Fig. 2). There was no radiological evidence of cholecystitis.

While the patient had no signs or symptoms concerning for ascending cholangitis, given the calcified sludge within the biliary tree the patient was deemed high risk for cholangitis in the context of biliary obstruction. The patient was therefore started on intravenous piperacillin/tazobactam prophylactically before intervention.

**Figure 3:**
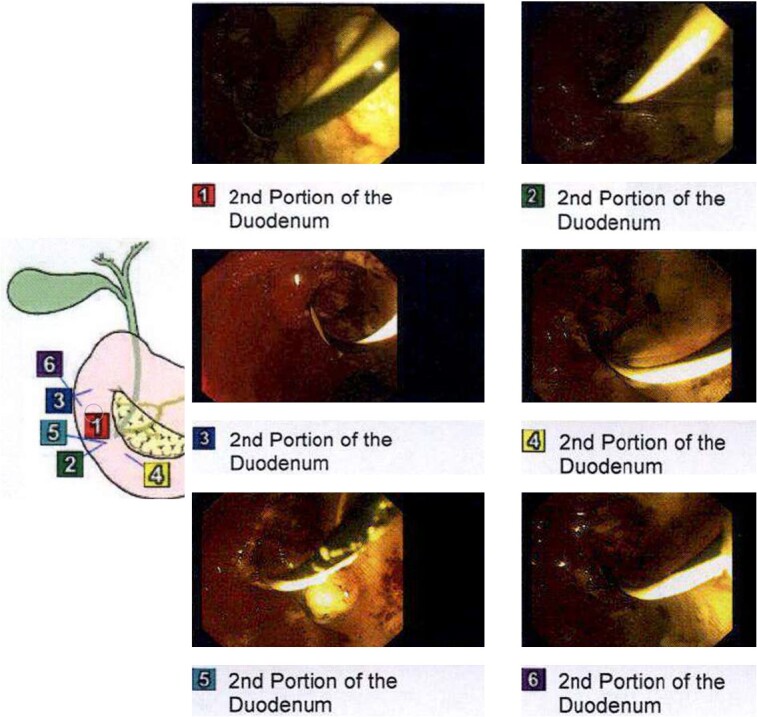
Intraprocedural images during ERCP demonstrating chalky limy bile.

Endoscopic retrograde cholangiopancreatography (ERCP) was performed on day three of admission. Following CBD cannulation and sphincterotomy, balloon trawl of the duct was performed yielding a large volume of chalky, granular material with detritus and stones; confirming a diagnosis of LBS (Fig. 3). A stent however could not be placed as the gravelly material blocked the channel of the endoscope. Therefore, the obstructing calculus in the distal CBD was not obviously removed hence the recommendation was for intraoperative cholangiogram (IOC) to be performed at laparoscopic cholecystectomy to confirm if a filling defect remained in keeping with a calculus.

**Figure 4:**
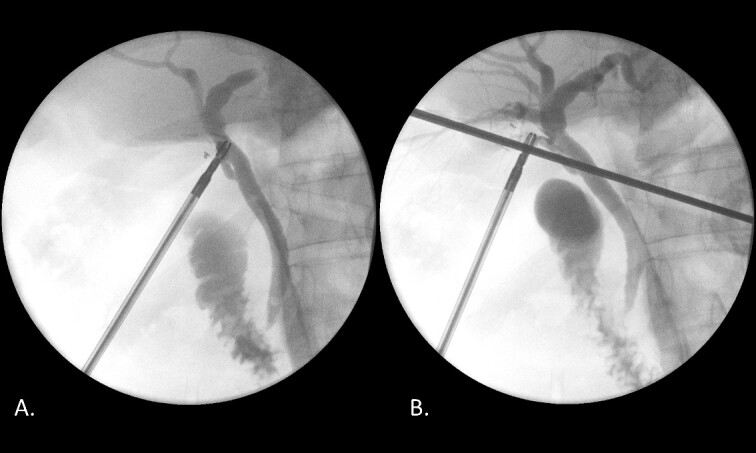
(A) IOC demonstrating a distal filling defect in the CBD causing partial occlusion. (B) IOC following TCBDE demonstrating no remaining filling defect with tapering of the distal CBD with contrast flow to the duodenum.

Laparoscopic cholecystectomy, IOC and transcystic bile duct exploration (TCBDE) were performed two days later. Intraoperatively, the gallbladder did not appear inflamed however there were signs of chronic inflammation with fibrosis within the hepatocystic triangle. An IOC demonstrated a distal CBD filling defect with flow into the duodenum suggesting persistent partial obstruction (Fig. 4). A TCBDE was performed with extraction of soft chalky sludge from the cystic duct. A completion IOC demonstrated no ongoing filling defect with flow to the duodenum and intrahepatic ducts. Histopathological sections of the gallbladder demonstrated findings compatible with chronic cholecystitis without evidence of dysplasia or malignancy.

Antibiotics were ceased and the patient was discharged the following day. At the routine 4-week follow up, the patient had resolution of symptoms, and his LFTs were within normal range.

## Discussion

The pathophysiological mechanism behind the development of LBS remains elusive without a clear cause being identified in the literature. It has been postulated obstruction of the cystic duct or gallbladder is a requirement, resulting in stasis of bile and changes in the local chemical environment of the gallbladder [1]. This is supported by the strong association of limy bile with the presence of cholelithiasis [1]. The role of metabolic calcium dysregulation on a systemic level has been proposed in the past and there exists at least two case reports in the literature of LBS presenting with concurrent hyperparathyroidism [2, 3]. As noted, parathyroid hormone and calcium levels were normal in our patient.

Often found histologically, chronic inflammatory changes in the gallbladder are thought to be secondary to an obstructive process or cholelithiasis rather than due to the limy bile itself [1, 4]. Chronic inflammation coinciding with limy bile is significant given the malignant potential chronic cholecystitis carries [5, 6]. While limy bile itself is not thought to be premalignant and there are no published case reports linking limy bile to malignancy, clinicians should be aware of these changes within the gallbladder [7].

The primary differential for hyperdense material in the biliary tract is intrabiliary contrast media. Cholangiographic contrast media such as intravenous meglumine iotroxate used in CT cholangiography is used purposefully to opacify the biliary tract. Recent cholangiographic procedures such as ERCP or IOC may also account for opacification of biliary anatomy on CT imaging. Traditional intravenous CT contrast media may also be excreted in bile via vicarious contrast excretion. First recorded in 1982, the phenomenon is not widely known outside of radiology and is most often seen in patients with renal impairment although it has also been reported as a normal variant [8].

Given the theoretical malignant potential associated with chronic cholecystitis, it has been advocated by at least one author that early intervention is warranted even in the absence of symptomatic disease [5]. Unfortunately, no guidelines exist in the management of LBS although cholecystectomy has formed the basis of treatment in symptomatic disease.

In our case, ERCP was performed first given the perceived risk of cholangitis followed by laparoscopic cholecystectomy, IOC and TCBDE performed 2 days later to ensure clearance of the CBD and prevent recurrence. As always, patient factors, disease severity and availability of interventions, such as ERCP, influence the management.
